# Insertion of Vaccinia Virus *C7L* Host Range Gene into NYVAC-B Genome Potentiates Immune Responses against HIV-1 Antigens

**DOI:** 10.1371/journal.pone.0011406

**Published:** 2010-06-30

**Authors:** José Luis Nájera, Carmen Elena Gómez, Juan García-Arriaza, Carlos Oscar Sorzano, Mariano Esteban

**Affiliations:** 1 Department of Molecular and Cellular Biology, Centro Nacional de Biotecnología, CSIC, Ciudad Universitaria Cantoblanco, Madrid, Spain; 2 Biocomputing Unit, Centro Nacional de Biotecnología, CSIC, Ciudad Universitaria Cantoblanco, Madrid, Spain; The University of Chicago, United States of America

## Abstract

**Background:**

The highly attenuated vaccinia virus strain NYVAC expressing HIV-1 components has been evaluated as a vaccine candidate in preclinical and clinical trials with encouraging results. We have previously described that the presence of *C7L* in the NYVAC genome prevents the induction of apoptosis and renders the vector capable of replication in human and murine cell lines while maintaining an attenuated phenotype in mice.

**Methodology/Principal Findings:**

In an effort to improve the immunogenicity of NYVAC, we have developed a novel poxvirus vector by inserting the VACV host-range *C7L* gene into the genome of NYVAC-B, a recombinant virus that expresses four HIV-1 antigens from clade B (Env, Gag, Pol and Nef) (referred as NYVAC-B-C7L). In the present study, we have compared the *in vitro* and *in vivo* behavior of NYVAC-B and NYVAC-B-C7L. In cultured cells, NYVAC-B-C7L expresses higher levels of heterologous antigen than NYVAC-B as determined by Western blot and fluorescent-activated cell sorting to score Gag expressing cells. In a DNA prime/poxvirus boost approach with BALB/c mice, both recombinants elicited robust, broad and multifunctional antigen-specific T-cell responses to the HIV-1 immunogens expressed from the vectors. However, the use of NYVAC-B-C7L as booster significantly enhanced the magnitude of the T cell responses, and induced a more balanced cellular immune response to the HIV-1 antigens in comparison to that elicited in animals boosted with NYVAC-B.

**Conclusions/Significance:**

These findings demonstrate the possibility to enhance the immunogenicity of the highly attenuated NYVAC vector by the insertion of the host-range gene *C7L* and suggest the use of this modified vector as an improved vaccine candidate against HIV/AIDS.

## Introduction

AIDS is one of the greatest pandemics of our time, affecting the health and the social and economic foundations of countries worldwide. An effective human immunodeficiency virus (HIV) vaccine offers the best hope for controlling the spread of the virus. While the immune correlates of protection are not well defined, both antibodies and T-cell responses contribute to control the infection with HIV or the related simian immunodeficiency virus (SIV), as well as disease progression [1], [2], [3], [4], [5]. Appropriate designed envelope immunogens able to induce broad and potent neutralizing antibodies remained a major goal for vaccine development and hence, vaccines directed to elicit virus specific cellular immune responses have been more readily developed, but their role in protection remains to be established. In this regard the recent observations of limited protection against HIV-1 infection, about 31%, in a phase III Thai clinical trial with a combination of a recombinant canarypoxvirus and the protein gp-120, points in the direction that both humoral and cellular immune responses might be needed for protection against HIV/AIDS, although the specific T cell and neutralizing antibody responses in the trial were low [6]. These clinical findings highlight that poxvirus vectors should be considered as one of the future HIV/AIDS vaccine candidate vectors, but that further vector development is needed.

Indeed, poxvirus vectors have emerged as prominent vehicles for delivering antigens of HIV-1. Different strains of Vaccinia Virus (VACV) expressing HIV-1 antigens such as Env, Gag, Pol and Nef or other components of HIV-1 have been evaluated in non-human primate [7], [8], [9], [10] and human trials [11], [12], [13]. While most of these recombinant viruses do not produce virus progeny in human cells, which assures safety, they are generally not potent HIV-1 immunogens by themselves and required priming with other vectors, such as DNA, to enhance the immune responses to HIV-1 antigens in animal models [14] and humans [12]. NYVAC and MVA are promising highly attenuated VACV vectors [15], [16], that in a head-to-head comparison in macaques elicited similar levels of protection after a challenge with SHIV_89.6P_,[9]. In a phase I clinical trial, the combination of recombinant DNA prime/NYVAC boost regimen (with both vectors expressing Env, Gag, Pol and Nef of HIV-1 from clade C) revealed that this vaccination approach was highly immunogenic, eliciting potent, broad, polyfunctional, and durable T-cells responses in 90% of vaccinees [13]. Since the protocol of DNA/NYVAC induced a greater CD4^+^ T cell response over CD8^+^ T cells and immunodominance for Env over Gag-Pol-Nef antigens, it suggest that to obtain a more balanced response to HIV-1 antigens with the DNA/NYVAC immunization protocol, further improvements of the NYVAC vector are desirable. One way to achieve this goal might be through genetic modifications of the NYVAC vector.

NYVAC was derived from Copenhagen strain by the precise deletion of 18 open reading frames encoding functions involved in the pathogenicity of the virus as well as in host-range regulatory functions governing the replication competency of the virus on cells derived from certain species, including human and mouse [17]. By reintroduction of the VACV *C7L* host range gene [18] into NYVAC vector, the capacity of the virus to replicate in human and rodent cells was restored, while maintaining an attenuated phenotype in mice [19]. Replication-competent recombinant VACV-based vaccines have received increased attention. To date, several replication-competent recombinants based on VACV viruses have been used as vaccine vectors for many infectious diseases, demonstrating that they are able to elicit potent humoral and cell mediated immune responses, and they are able to confer lasting protection while maintaining a safe phenotype [20], [21], [22], [23]. In fact, a phase I vaccine trial in China has recently begun using attenuated, replication competent Tiantan VACV vector expressing multiple HIV-1 genes (Wen, J. Phase I study of China's compound HIV/AIDS vaccine begins. http://www.asia-lifesciences.com, January 10, 2008).

To improve the immunogenicity of the NYVAC based HIV/AIDS vaccine candidate, we have developed a novel vector by inserting the host range gene VACV *C7L* under the control of the synthetic early/late virus promoter into NYVAC-B, a recombinant virus which expresses four HIV-1 antigens from clade B (Env, Gag, Pol and Nef). Previously we showed that insertion of *C7L* into NYVAC genome prevented virus-induced apoptosis and the phosphorylation of the translational initiation factor eIF-2 alpha, allowing virus multiplication in human cells while the virus remains attenuated in infected mice [19]. Here, we have characterized the magnitude, breath, durability and quality of anti-HIV-1 immunity elicited by the replication competent NYVAC-B-C7L in comparison to the replication restricted NYVAC-B. Our findings revealed that NYVAC-B-C7L is superior to NYVAC-B for induction of specific cellular immune responses against HIV-1 antigens. Prime/boost with DNA-B/NYVAC-B-C7L-induced T-cell responses mediated by both CD4^+^ and CD8^+^ T cells, and these responses are multifunctional, persistent, durable and directed to the different vaccine-encoded HIV-1 antigens

## Results

### Generation and characterization of recombinant NYVAC-B-C7L in infected human cells

**Figure 1:**
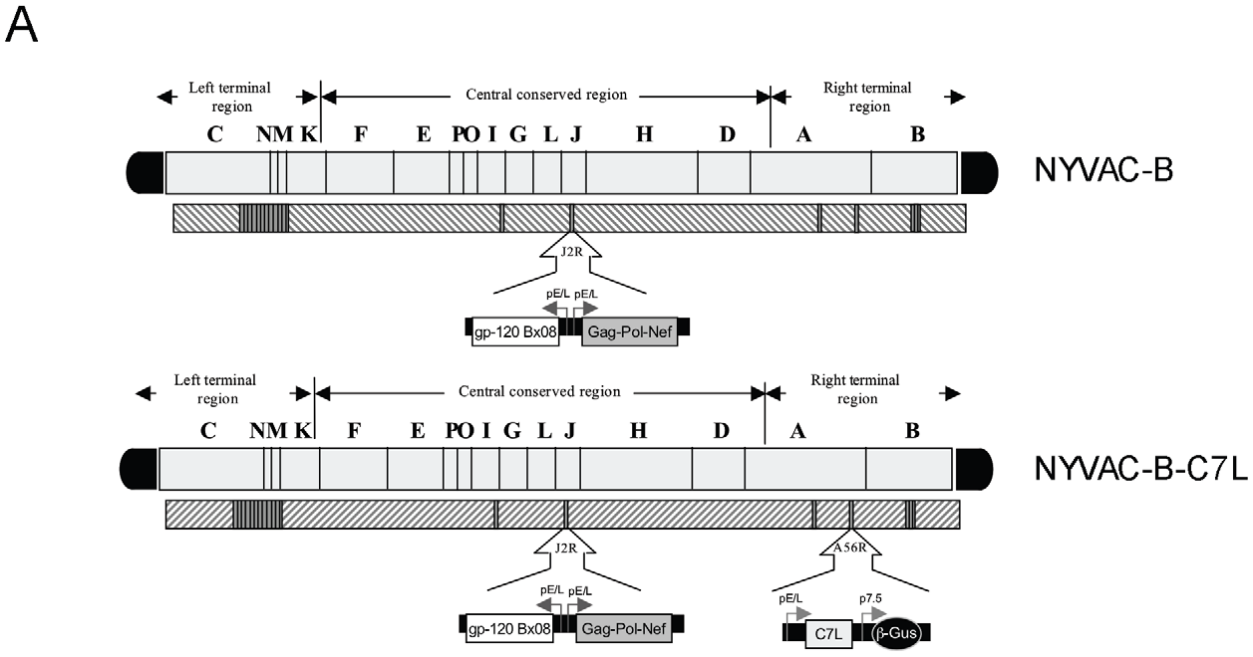
Diagram of the genome of NYVAC-B and NYVAC-B-C7L recombinant vectors. **A.** Diagram of the two vectors with the genes encoding HIV-1 antigens inserted in the TK locus of the viral genome and with *C7L* at the HA locus.

In an effort to further improve the immunogenicity of NYVAC based vaccine candidates against HIV/AIDS, we have generated a new vector by inserting the VACV *C7L* host range gene in the attenuated NYVAC-B recombinant which expresses four HIV-1 antigens from clade B (Env, Gag, Pol and Nef). As shown in Figure 1A, NYVAC-B-C7L contains the *C7L* gene inserted into the HA locus (*A56R*) of NYVAC-B genome under the transcriptional control of the synthetic early/late (E/L) virus promoter and preserves, as its parental NYVAC-B, the HIV-1 genes inserted in the TK locus (*J2R*). The correct insertion of heterologous genes in the recombinant virus as well as the purity of the stocks was confirmed by PCR (data not shown).

**Figure 2:**
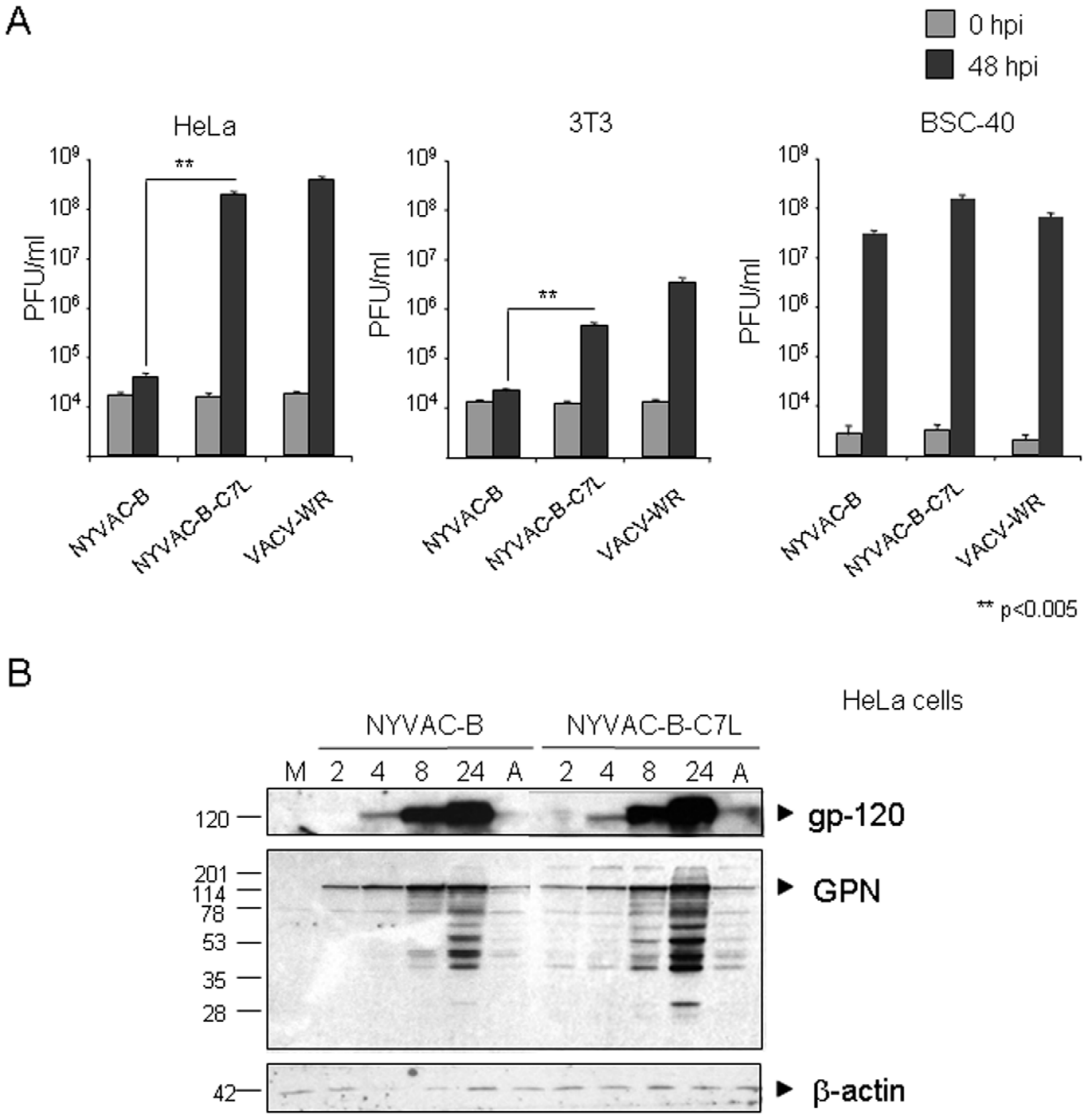
Viral growth efficiency and expression of HIV-1 antigens by NYVAC-B and NYVAC-B-C7L vectors. **A.** HeLa, 3T3 and BSC40 cells were infected with NYVAC-B, NYVAC-B-C7L or VACV-WR at 0.01 PFU per cell and at 48 hpi cells were collected with the media. The cells were frozen and thawed three times, sonicated and virus infectivity was titrated by plaque assay in BSC-40 cells. Data are presented as the mean ± SD. **B.** Western blot showing the kinetics of expression of gp-120 and GPN with time of infection. HeLa cells were infected with 5 PFU/cell in the absence or presence of AraC (A), cell extracts collected at various times, analyzed by SDS-PAGE, and Western blots reacted with specific antibodies to Env and GPN. Actin was used as a loading control. M: uninfected mock cells.

The functionality of *C7L* gene was determined by measuring the viral growth efficiency of the recombinant viruses in three different cell lines of human (HeLa), mouse (3T3) and monkey (BSC40) origin. Monolayers of cells were infected at 0.01 PFU/cell with NYVAC-B or NYVAC-B-C7L, and at 0 and 48 hours postinfection the cells were collected with the media and virus titers in cell homogenates were determined by plaque assay. As shown in figure 2A, insertion of C7L gene in NYVAC-B genome renders the vector capable of replication in human and murine cell lines. The yields of NYVAC-B-C7L in HeLa cells were similar to those produced by replication-competent VACV-WR strain or in permissive BSC-40 cells. In mouse cells (3T3), while NYVAC does not produce progeny virus, NYVAC-B-C7L also replicates similarly as VACV-WR but the virus yields were 1 to 2 logs lower than in infected HeLa cells. The presence or absence of C7L gene has no effect on NYVAC-B replication in BSC-40 cells.

We have previously observed that NYVAC-B expresses efficiently the HIV-1 proteins, _Bx08_gp-120 as a protein secreted from cells and _III-B_GPN as an intracellular fusion polyprotein of about 150 kDa [24]. To compare the expression levels of the HIV-1 proteins by NYVAC-B and NYVAC-B-C7L, a time course was carried out in infected HeLa cells. As shown in the Western blot of Figure 2B, the kinetics of synthesis of HIV-1 proteins were similar in cells infected with both viral recombinants. However, the _Bx08_gp-120 and _III-B_GPN expression levels at late times post-infection were higher in cells infected with NYVAC-B-C7L than in cells infected with NYVAC-B. The protein expression levels were similar when infected cells were treated with the DNA synthesis inhibitor Ara C, suggesting that differences in antigen expression between both viral recombinants are mainly a late event. Similar results for enhanced expression of gp120 and of GPN by NYVAC-B-C7L were observed in infected monkey BSC40 and mouse 3T3 cells (Fig. S1, supplementary data).

**Figure 3:**
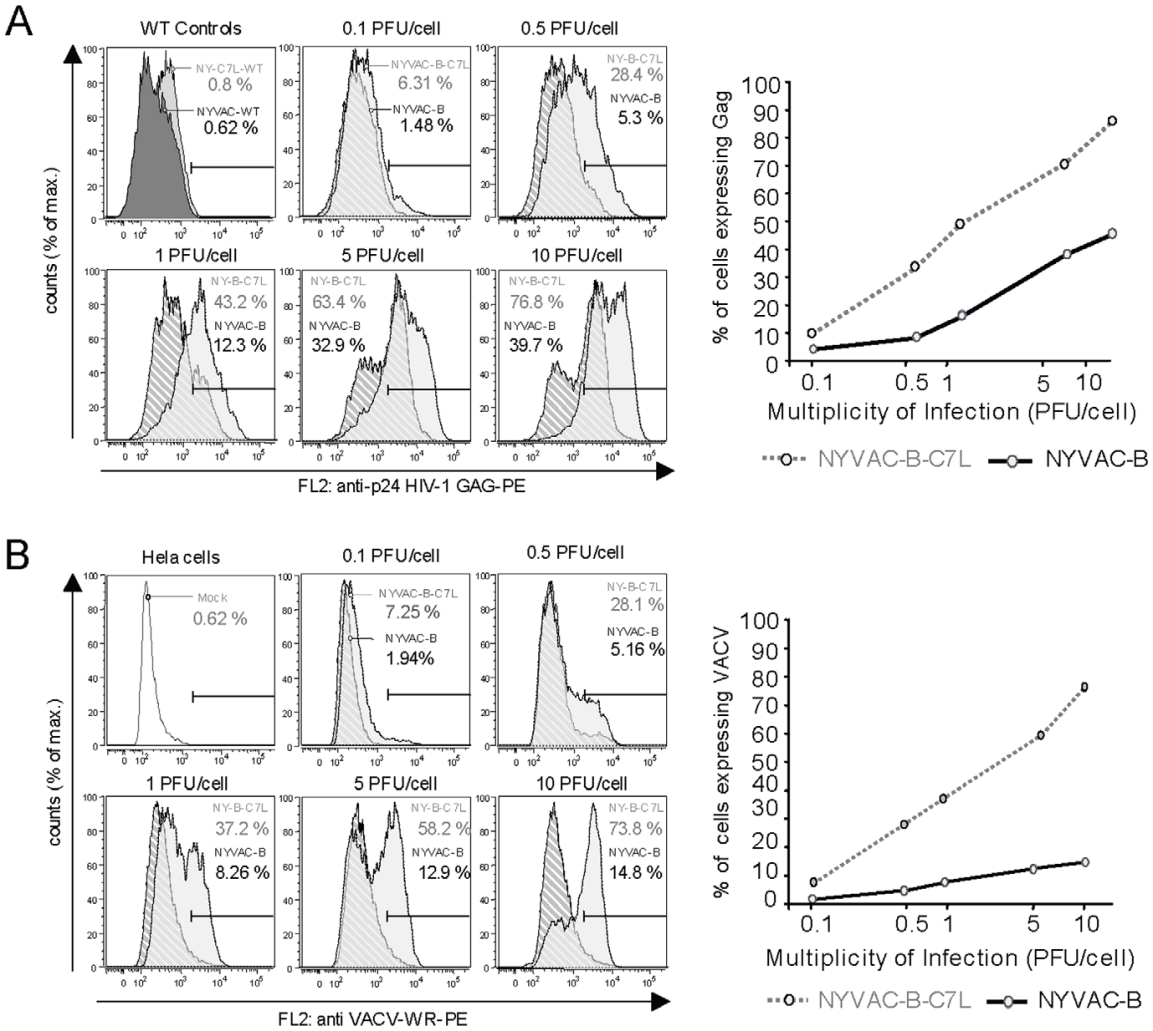
FACS analysis of HIV-1 Gag and VACV antigens expression. HeLa cells were infected with different MOIs of NYVAC-B or NYVAC-B-C7L, and cells were processed for FACS after reactivity with antibodies to HIV-1 p24 for Gag (**A**) or anti-VACV for virus expression (**B**), as described under Materials and Methods. The doses of virus used to infect HeLa cells are given above FACScans. The extent of expression by NYVAC-B is denoted by the black curve, while NYVAC-B-C7L expression is denoted by the grey curve. Dose-response curves for expression of vaccine insert vs. titer of input virus are shown in the right panels.

To analyze in more detail the differences in expression between NYVAC-B and NYVAC-B-C7L recombinants, we performed a FACS analysis to quantify the production of HIV-1 and VACV proteins. HeLa cells were infected with different viral doses of the two recombinants or with the parental viruses (NYVAC-WT and NYVAC-C7L). At 18 hours post-infection, cells were harvested, stained for HIV-1 p24 Gag or for late structural VACV proteins with the corresponding polyclonal antibodies, and we analyzed the frequency of Gag and VACV expressing cells as described in Materials and Methods. The FACS analysis revealed that the presence of *C7L* in NYVAC-B genome increased by 2- to 5.3- fold the percentage of cells expressing HIV-1 p24 Gag (Figure **3A**) or VACV antigens (Figure 3B) in comparison to NYVAC-B. The maximum differences in HIV-1 p24 Gag expression were observed at 0.1 PFU/cell (4.3 fold) and 0.5 PFU/cell (5.3 fold). We hypothesize that this is because at low viral doses not all cells become infected by NYVAC-B, while NYVAC-B-C7L was able to replicate and spread leading to a higher number of cells expressing the heterologous antigen. At higher MOIs of 5 and 10 PFU/cell the increase was only two-fold, as all cells are infected with both viruses. After NYVAC-B-C7L infection, there was a near proportional increase as a function of the MOI in HIV-1- and VACV- expressing cells compared with NYVAC-B infection (Figure 3A and 3B). The reduced reactivity of the antibodies to VACV in NYVAC-B infected cells reflects decreased levels of late viral antigens in these cells compared with cells infected with NYVAC-B-C7L, an indicator of enhanced virus multiplication as a result of the presence of *C7L* gene. We have previously shown a blockade of certain late viral proteins in HeLa cells infected with NYVAC [19]. These findings suggest that *C7L* not only allowed NYVAC-B-C7L to replicate in HeLa cells but also improved the production of HIV-1 antigens in infected cells.

### Viral distribution of NYVAC-C7L in tissue organs after systemic infection

**Figure 4:**
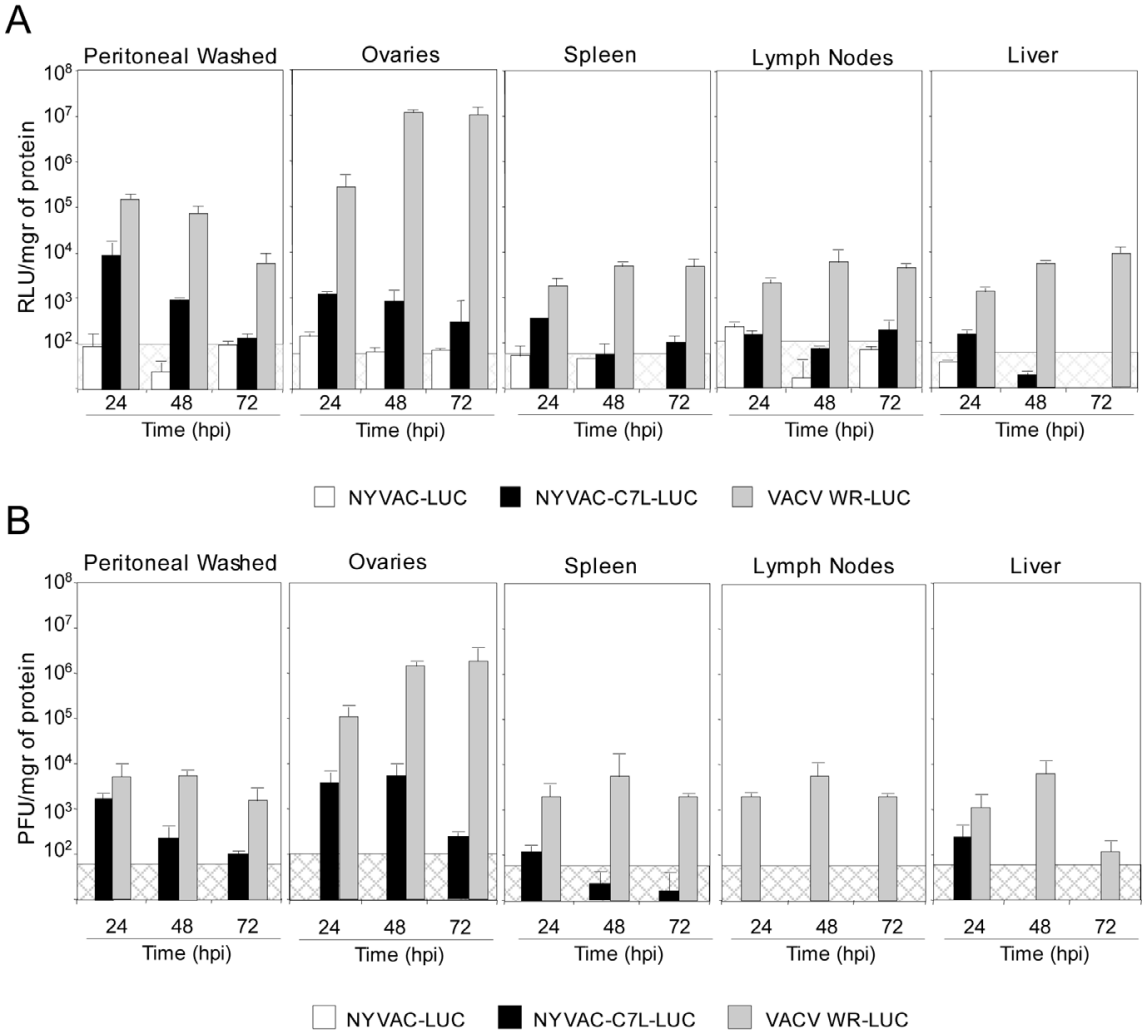
Kinetics of luciferase expression and of viral titers in mice infected with NYVAC-Luc or NYVAC-C7L-Luc. BALB/c mice were inoculated i.p. with 2×10^7^ PFU/mouse of WR-Luc, NYVAC-Luc or NYVAC-C7L-Luc. Tissues were collected at different times post-infection and both luciferase expression and virus titers determined as described under Materials and Methods. Results represent mean values from samples of three animals per time and grouped with standard deviation of three independent experiments. Background levels in control, uninfected tissue are shown as dashed boxes. **A.** Kinetics of luciferase expression with time expressed as RLU per mg of tissue evaluated by luciferase assay. **B.** Kinetics of virus titers with time expressed as PFU per mg of tissue evaluated by plaque assay.

We have previously reported the use of the luciferase marker to follow VACV replication and antigen expression in mice [25], [26]. To determine whether the insertion of the *C7L* gene into NYVAC genome affects the virus distribution and heterologous antigen expression *in vivo*, BALB/c mice were inoculated i.p. with a dose of 2×10^7^ PFU of NYVAC-Luc, NYVAC-C7L-Luc or WR-Luc which is one of the most effective routes for systemic virus dissemination [26]. At 24, 48 and 72 h post-inoculation, animals were sacrificed and the luciferase activity and virus production in different tissues were assayed in cell homogenates derived from peritoneal cavity, ovaries, spleen, lymph nodes and liver. While luciferase activity was clearly observed in all tissues from WR-Luc infected mice, in comparison NYVAC-Luc expresses greatly reduced levels of luciferase. Insertion of *C7L* gene into the NYVAC genome allows the recombinant virus to express luciferase to levels higher than the parental virus, mainly in peritoneal cells, ovaries and spleen (Figure 4A). The results on luciferase expression, in general, correlated well with virus titers in the different tissues (Figure 4B). Although the values observed in the lymph nodes at 24 hpi are quite low, close to the cut-off baseline, a direct correlation between luciferase and PFU can not be made in this tissue. Only in those samples giving positive values of luciferase and PFU production, a correlation of luc expression and of virus progeny can be made. The results of viral biodistribution in mice were performed at least three times with similar results. Although NYVAC-C7L-Luc was able to replicate to limited extent after 3 days of infection, its replication capacity was restricted, probably because it still lacks other host range and immunomodulatory genes in its genome and as a consequence the virus was clarified faster than WR-Luc.

Together, these results revealed that NYVAC-C7L maintains a restricted replication phenotype in mouse tissues while it expresses higher levels of heterologous antigen than parental NYVAC. The enhanced levels of expression of NYVAC-C7L could provide the vector with a more effective capacity than parental NYVAC to induce stronger specific immune response to HIV-1 antigens.

### Immunogenicity of NYVAC-B-C7L during DNA prime/poxvirus boost vaccination

Since the timing of antigen exposure has been shown to influence the magnitude and quality of the T-cell response, we next analyzed the specific HIV-1 immune responses elicited in mice by NYVAC-B and NYVAC-B-C7L recombinants using a DNA prime/Pox boost approach.

**Figure 5:**
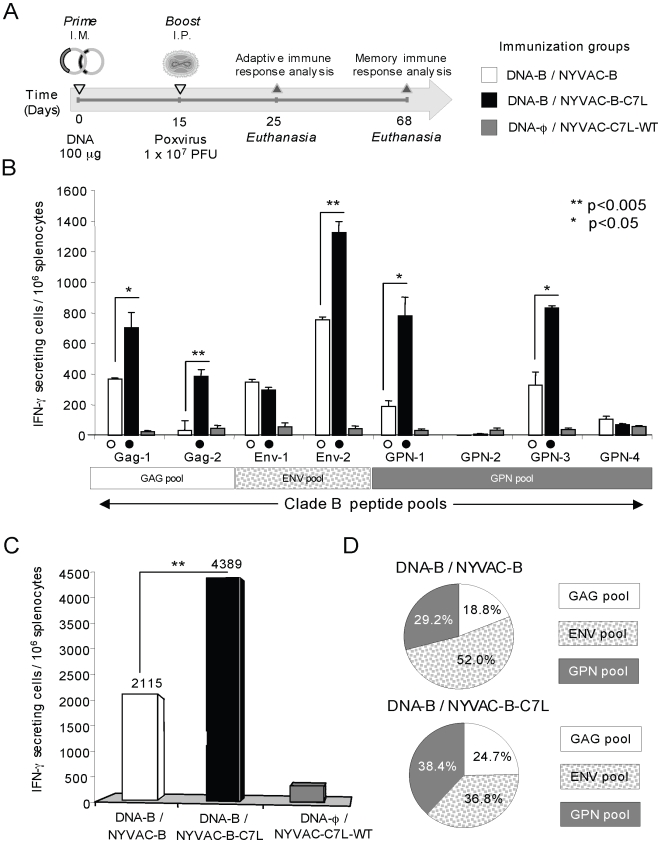
Immunogenicity of NYVAC-B versus NYVAC-B-C7L. **A**) Immunization schedule. Eight BALB/c mice received 100 µg of DNA-B (50 µg of pCMV-_BX08_gp-120+50 µg of pcDNA-_IIIB_GPN) by intramuscular route (i.m.) and two weeks later received an intraperitoneal (i.p.) inoculation dose of 1×10^7^ PFU of NYVAC-B or NYVAC-B-C7L in 200 µl of PBS. Ten days after the last immunization some of the mice (n = 4) were sacrificed and spleens processed for ELISPOT assay and Intracellular Cytokine Staining (ICS) to analyze adaptive immune response. At day 53 the remaining animals in each group (n = 4) were sacrificed and spleen processed for ICS to analyze the long-lived specific response. **B**) Cellular responses against HIV-1 antigens as measured by ELISPOT. Bars show mean values of the number of IFN-γ secreting splenocytes from mice immunized groups for each HIV-1 specific peptide pools that span the four HIV-1 antigens, and error bars represent the standard deviation. Filled symbols represent significant differences between each peptide pool and the negative control. * Represent statistically significant differences between groups. **C**) Magnitude of the total response for clade B pools. Bars represent the total number of antigen-specific IFN-γ secreting cells detected in each group against all the peptide pools spanning the HIV-1 antigens included in the recombinants. **D**) Mean proportion of the response directed to each of the HIV-1 vaccine antigens in both immunization groups. All the values are shown as net percentage of total HIV-1 specific IFN-γ secreting cells.

BALB/c mice, 4 in each group, were immunized according to the schedule shown in the diagram of Figure 5A. Animals received by i.m. route a dose of 100 µg of DNA-B (50 µg of pCMV- _Bx08_gp-120 + 50 µg of pcDNA _III-B_GPN) followed by an i.p. injection of 1×10^7^ PFU of NYVAC-B or NYVAC-B-C7L. Adaptive immune responses were measured 10 days after final immunization using a fresh IFN-γ ELISPOT assay with splenocyte stimulation using pools of overlapping peptides that span the HIV-1 antigens included in the immunogens.

As shown in Figure 5B, animals that received NYVAC-B or NYVAC-B-C7L in the booster, induced a significant enhancement of splenic T-cell responses against the clade B peptide pools Gag-1, Gag-2, Env-1, Env-2, GPN-1 and GPN-3 in comparison with animals from control group (DNA-φ/NYVAC-C7L-WT) (p<0.05). However, the highest response was detected in mice boosted with NYVAC-B-C7L. After DNA-B/NYVAC-B-C7L immunization, positive responses were observed against six of the eight HIV-1 peptide pools assayed while after NYVAC-B immunization, five out of eight pools were considered HIV-1-specific. The improvement induced by NYVAC-B-C7L was observed in five of those HIV-1 peptide pools (Figure 5B). The number of IFN-γ secreting cells increased from 2- to 10- fold in GAG pool, nearly 2-fold in ENV pool, and between 2- to 4- fold in GPN pool as compared to DNA-B/NYVAC-B.

Similarly, the magnitude of the total HIV-1 response (Figure 5C), determined by the overall number of IFN-γ secreting cells, was significantly higher when NYVAC-B-C7L was used as a booster in comparison to that elicited by DNA-B/NYVAC-B regimen (4389 versus 2115 SFU per 10^6^ splenocytes, p<0.005).

Interestingly, when calculating the average proportion of the response to the HIV-1 vaccine-expressed genes (Figure 5D), it was evident that the response elicited in animals immunized with DNA-B/NYVAC-B was dominated by Env antigen (more than 50% of the total HIV-1 response), as we have previously described [9], [24]. However, the distribution of the HIV-1 specific immune response after boosting with NYVAC-B-C7L was more balanced and directed towards the three antigens similarly (ENV: 36.8%; GAG: 24.7% and GPN: 38.4%) suggesting that with this regimen, the response was not dominated by a single vaccine antigen. Similar findings were observed in three independent experiments.

Overall, the combination of a DNA-B prime followed by the NYVAC-B-C7L vaccine construct boost induced a more balanced, broader, and higher IFN-γ HIV-1-specific T-cell responses as compared with that elicited by a DNA-B/NYVAC-B immunization protocol.

### Functional profile of NYVAC-B and NYVAC-B-C7L induced CD4^+^ and CD8^+^ T-cell responses

**Figure 6:**
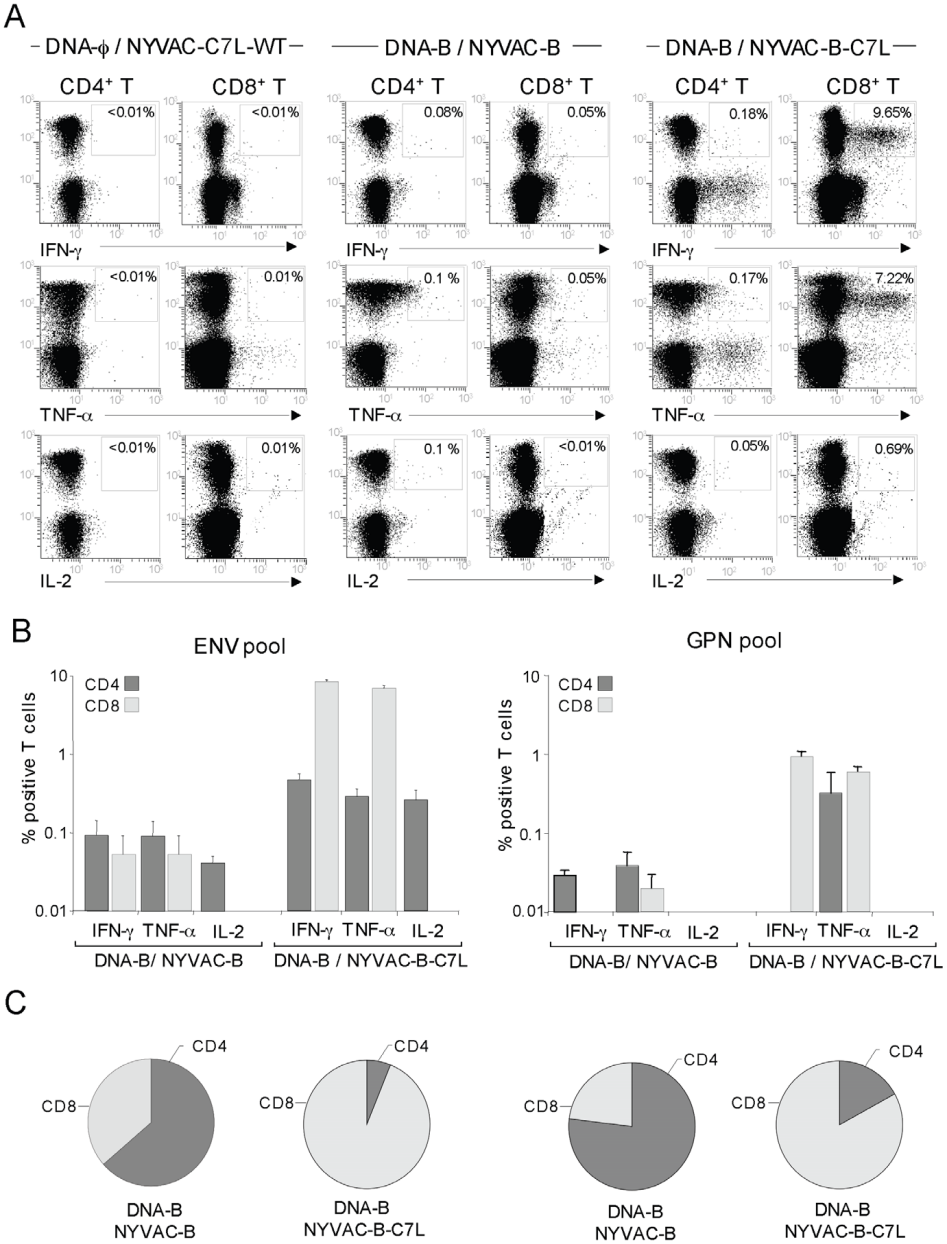
Phenotypic analysis of vaccine-induced CD4^+^ and CD8^+^ T-cell responses. Splenocytes obtained ten days post-immunization from animals vaccinated with DNA-B plus NYVAC-B or NYVAC-B-C7L were used to analyze HIV-1-specific CD4^+^ and CD8^+^ T cell functions after stimulation with the same peptide pools as for ELISPOT using polychromatic flow cytometry. Cells were gated by forward and side scatter, CD3^+^ T cells and then CD4^+^ and CD8^+^ T cells. **A**) Representative flow cytometry plots. The numbers indicate the percentage of CD8^+^ or CD4^+^ T cells expressing cytokine(s) IFN-γ, IL-2 and/or TNF-α. In the figure only HIV-1 specific CD8^+^ and CD4^+^ T cells stimulated *ex vivo* with ENV peptide pool are shown. **B**) Percentages of CD4^+^ and CD8^+^ T cells secreting IFN-γ, TNF-α or IL-2 in each immunization group after stimulation with HIV-1 Env pool or GPN pool. **C**) The pie chart summarizes the data and each slice of the pie corresponds to the fraction of CD4^+^ or CD8^+^ T cells specific for HIV-1 antigens within the total CD4^+^ or CD8^+^ T populations.

To determine the phenotypic characteristics of the T-cell populations activated after immunization with the DNA/Pox combination, we employed multiparameter intracellular FACS analysis to identify the HIV-1-specific T-cell responses. Splenocytes were cultured overnight and then stimulated with ENV, GAG or GPN peptide pools for 6 hours in the presence of brefeldin A. Representative functional profiles from splenocytes of animals vaccinated with DNA-B followed by NYVAC-B or NYVAC-B-C7L stimulated with ENV pool are shown in Figure 6A. A control group corresponding to animals vaccinated with sham DNA plus NYVAC-C7L-WT was also included in the study. The panel of T-cell functions we analyzed included IFN-γ, TNF-α and IL-2 secretion for both CD4^+^ and CD8^+^ T cells. As illustrated in Figure 6, both immunization regimens induced antigen-specific IFN-γ, TNF-α and IL-2 responses against ENV and GPN HIV-1 antigens, but in NYVAC-B-C7L the frequency of HIV-1-specific cytokine responses were markedly increased. Comparative immunogenicity studies indicated a difference between groups of 2- to 200- fold in the percentage of HIV-1- specific CD4^+^ T and CD8^+^ T cells. For the GAG response the CD8+ T cell compartment was enhanced 4 fold in NYVAC-B-C7L in comparison to NYVAC-B (data not shown). With regard to ENV- and GPN-specific responses, NYVAC-B-boosted animals responded mainly to Env and Gag antigens, and the response was mediated by both CD4^+^ and CD8^+^ T-cell populations (Figures 6B and 6C). In contrast, in NYVAC-B-C7L boosted animals, the response was directed mainly against Env, Gag and GPN antigens and these T-cell-mediated HIV-1 specific responses were significantly higher than in the group of NYVAC-B and predominantly mediated by CD8^+^ T cell populations (Figures 6B and 6C). The CD8^+^ compartment was responsible for most (>90%) of the total IFN-γ and TNF-α responses and the IL-2 secretion was mainly mediated by CD4^+^ T cells in DNA-B/NYVAC-B-C7L group after ENV stimulation. In contrast, in the DNA-B/NYVAC-B immunization group, the CD4^+^ compartment was responsible for more than 60% of the production of the three cytokines.

**Figure 7:**
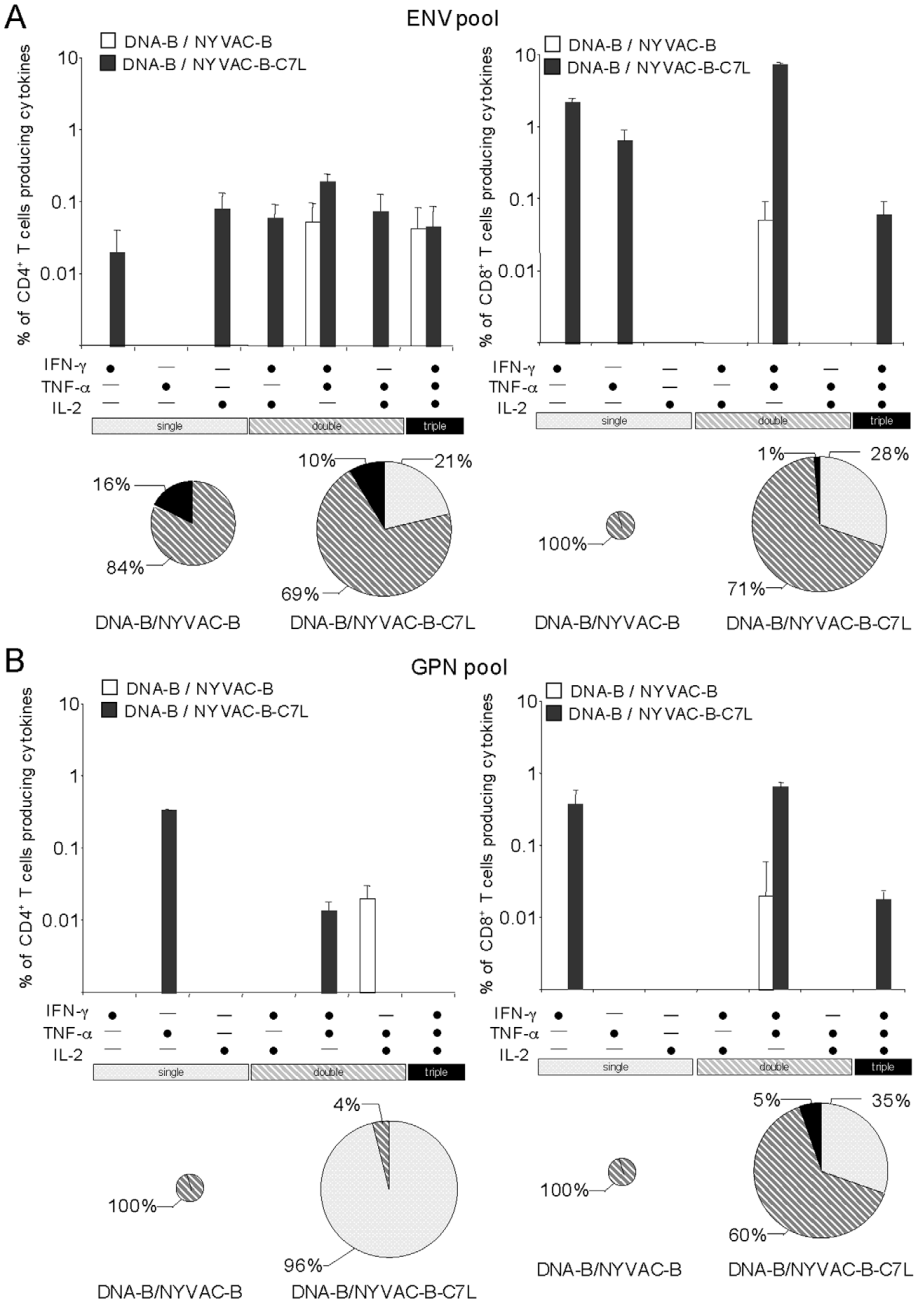
Functional profile of vaccine-induced CD4^+^ and CD8^+^ T cells. Functional composition of CD4^+^ and CD8^+^ T cells responses against ENV (A) and GPN (B) based on the secretion of IFN-γ, IL-2 and/or TNF-α. All the possible combinations of the responses are shown on the X axis, whereas the percentages of the functionally distinct cell populations are shown on the Y axis. Bars correspond to the fraction of different functionally distinct T-cell populations within total CD4^+^ and CD8^+^ populations. Responses are grouped and color-coded on the basis of the number functions. The pie chart summarizes the data and each slice of the pie correspond to the fraction of CD4^+^ or CD8^+^ T cells with a given number of functions within the total CD4^+^ or CD8^+^ T populations. The size of the pie chart represents the magnitude of the specific HIV-1 immune response induced.

The simultaneous measurements of three functions allowed the assessment of the quality of the vaccine-induced CD4^+^ and CD8^+^ T-cell responses. On the basis of the analysis of IL-2, IFN-γ and TNF-α secretion, seven distinct HIV-1 specific CD4^+^ and CD8^+^ T-cell populations were identified. To further characterize the immunogenicity triggered in each immunized group, we assessed polyfunctional T-cell responses. The ENV-specific CD4^+^ and CD8^+^ T cell responses elicited by DNA-B/NYVAC-B or DNA-B/NYVAC-B-C7L groups were polyfunctional, as more than 70% of CD8^+^ T cells and 80% of CD4^+^ T cells had two or three functions based on IFN-γ, TNF-α and IL-2 secretion. However, some differences were observed between both groups. GPN-specific CD8^+^ and CD4^+^ T-cell responses were highly polyfunctional in the DNA-B/NYVAC-B immunization group, while in DNA-B/NYVAC-B-C7L only CD8^+^ T cells were multifunctional. Examination of the relative contributions of individual cytokines versus polyfunctional responses showed that NYVAC-B-C7L improves the quality of the response in CD8^+^ T cell compartment but not in the NYVAC-B group, with a proportion (1–5%) of the HIV-1-specific CD8^+^ T cells expressing simultaneously the three cytokines,. In contrast, no improvement was found in the CD4^+^ T-cell compartment in terms of the quality of the response (Figure 7).

Overall, these results indicated that both immunization regimens induced polyfunctional ENV and GPN-specific T cell responses and demonstrated that DNA-B/NYVAC-B-C7L immunization improved the magnitude and quality of the anti-HIV-1 response compared to NYVAC-B.

### Polyfunctionality of long-lived HIV-1 specific T-cell responses induced by NYVAC-B-C7L

The longevity of the immune response generated by a vaccine may be critical for its success. In order to gain insights on the durability of the T-cell response generated in mice by the DNA/Pox immunization protocol outlined in Figure 5A, we analyzed the T cell profiling at 53 days post-vaccination. Functional characterization was performed from the measurements of CD4^+^ and CD8^+^ T cells secreting IFN-γ and/or IL-2 after Ag-specific stimulation.

**Figure 8:**
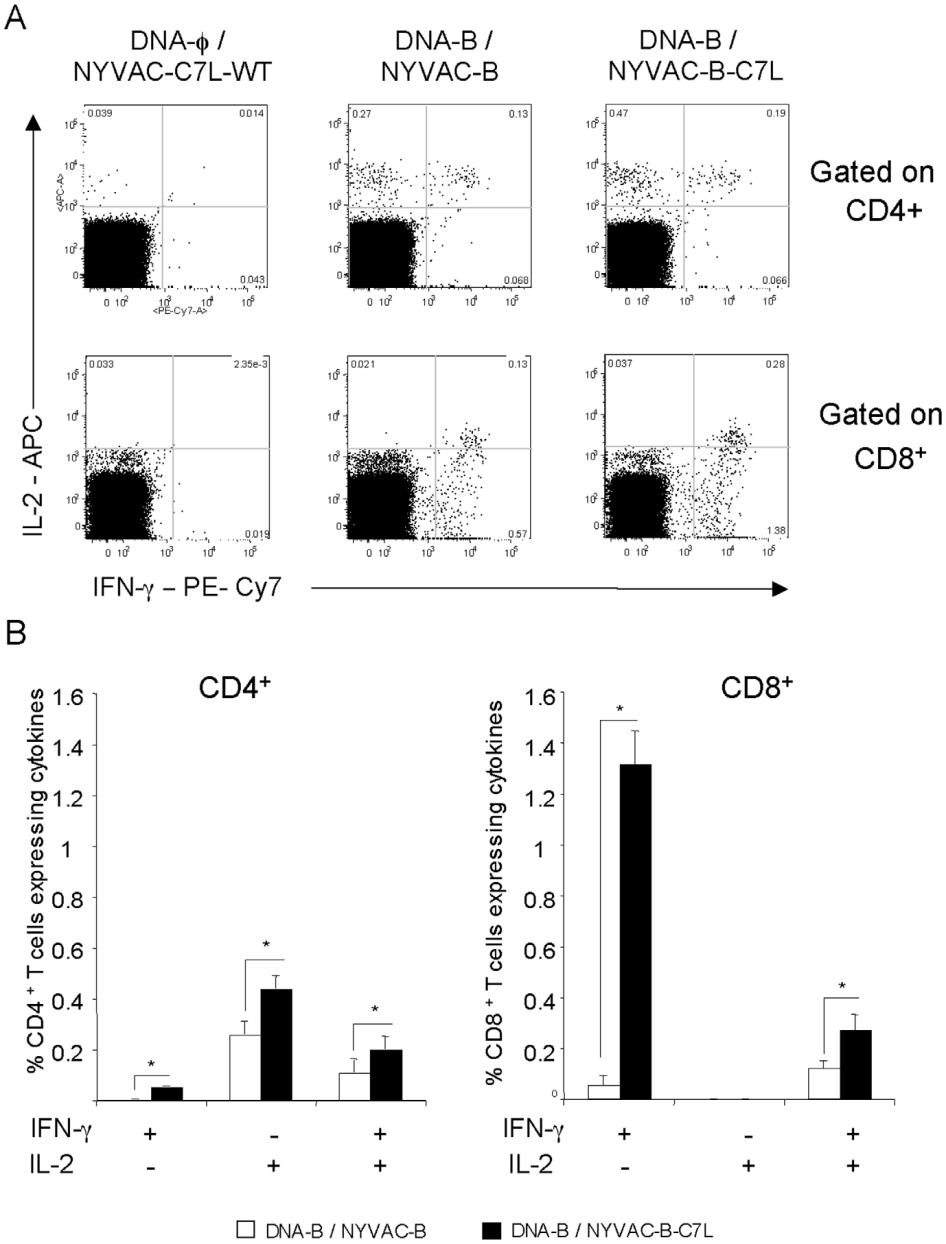
Long-lived responses to vaccination after ENV stimulation. Splenocytes obtained 53 days post- immunization from animals vaccinated with DNA-B plus NYVAC-B or NYVAC-B-C7L were used to analyze HIV-1-specific CD4^+^ and CD8^+^ T cell functions after stimulation with the same peptide pools used previously. Cells were gated by forward and side scatter, CD3^+^ T cells and then CD4^+^ and CD8^+^ T cells. In the figure only HIV-1 specific CD4^+^ and CD8^+^T cells stimulated *ex vivo* with ENV peptide pool are shown. **A**) Representative flow cytometry plots. The numbers indicate the percentage of CD4^+^ or CD8^+^ T cells expressing cytokine(s) IFN-γ and/or IL-2. **B**) Percentages of CD4^+^ and CD8^+^ T cells secreting IFN-γ and/or IL-2 in each immunization group after stimulation with HIV-1 ENV pool.

As shown in Figure 8 for CD8^+^ and CD4^+^ T cells, the NYVAC-B-C7L vector showed significantly higher Env-specific immune responses than NYVAC-B (2.25% versus 1.01%, p>0.05). While the Gag- and GPN-specific responses were also higher in NYVAC-B-C7L group, the differences were not statistically significant. For CD8^+^ T cells, higher levels of both IFN-γ and IL-2 were produced in mice primed with DNA-B and boosted with NYVAC-B-C7L than when boosted with NYVAC-B (2 fold). Interestingly, the production of IFN-γ by these cells was considerably higher than the production of IL-2. In fact, a high frequency of total IL-2 production by CD8^+^ T cells (more than 90%) resulted also positive for IFN-γ secretion representing 15% of the total Env-specific CD8^+^ T cell response. However, the Env-specific CD4^+^ T cells generated a higher frequency of IL-2 than IFN-γ response in both immunization groups and the percentage of multifunctional Env-specific CD4^+^ T cells was about 26% of the total response.

These findings demonstrate that both NYVAC-B and NYVAC-B-C7L induced long-lived HIV-1 specific T-cell responses but the magnitude and quantity of these responses were significantly enhanced by the use of NYVAC-B-C7L in the booster.

## Discussion

Different viral vectors of RNA and DNA origin are being considered as HIV/AIDS vaccine candidates, but to date only the poxvirus vector ALVAC in combination with a purified gp-120 prime boost has shown some efficacy in a phase III clinical trial [6]. Since ALVAC exhibits restricted replication in human cells, which limits the immune potency of the vector, other poxvirus vectors are sought as vaccine candidates. Among them, the attenuated strains of vaccinia virus MVA and NYVAC have been considered as promising HIV/AIDS vaccine candidates due to their strong safety record in preclinical and clinical trials, high stability, ability to induce potent and long-lasting immune responses to foreign antigens, and efficacy in immunized macaques after challenge with SIV [15]. Although MVA and NYVAC are safe vectors, their replication *in vivo* is limited which curtails the amount of antigen that can be produced in cells [26]. Thus, while vaccination with replication-restricted VACV recombinants is highly desirable for safety reasons, there might be an advantage in the use of replicating vectors eliciting prolonged immune responses. A close examination of licensed vaccines against infectious diseases reveals that some of the most potent and efficacious constructs currently in use are replicating vectors [27], [28]. Replication-competent recombinant VACV-based vaccines have received increased attention as potential vaccine vectors. To date, several replication-competent recombinant VACV-based vaccines have been used for various infectious diseases, demonstrating that they are able to elicit potent humoral and cellular mediated immune responses, and to confer lasting protection while maintaining a safety phenotype [20], [21], [22], [23]. A recent smallpox vaccine study has further emphasized the superiority of replication competent vectors to non-replicating in long term protection and immunogenicity against lethal cowpox challenge [29]. The need for replication competence of VACV vectors has been proposed as potential solution to the lack of efficacy of some of the highly attenuated vaccines that have been tested in human trials [20].

In an effort to improve the immune potency of the NYVAC vector, we have constructed a new vector with replication competence in human cultured cells. This was done by the insertion of VACV *C7L* gene into the genome of NYVAC-B, a vector that expresses gp-120 and Gag-Pol-Nef antigens of HIV-1 from clade B. The *C7L* gene was selected for insertion into NYVAC-B genome because it is a VACV host range gene [18] necessary for viral replication in human cells [30] and the viral product inhibits antiviral activities induced by type I interferons [31], [32]. Furthermore, we have previously described that *C7L* insertion into the parental NYVAC genome restores the viral replication cycle in cultured human and murine cells by preventing eIF2-α phosphorylation and the induction of apoptosis [19]. Here, we observed that the presence of *C7L* in the NYVAC-B genome improved significantly the level of expression of HIV-1 antigens as a result of enhanced translation, thus increasing viral replication and cell spreading. An increase in antigen expression observed in culture cells infected with NYVAC-C7L was also observed *in vivo*. As expected, the levels of luciferase activity were the highest in tissues from mice infected with WR-Luc, were moderate in NYVAC-C7L and the lowest in NYVAC-immunized mice, correlating luciferase levels with the replication capacity of each vector. We have previously described the safety profile of NYVAC-C7L, which remains replication competent in murine and humans cells but does not cause body weight loss or death after intranasal challenge in mice [19]. The limited virus replication observed in mouse tissues of NYVAC-C7L-Luc is probably related to the deletions contained in the viral genome that affect other host range and immunomodulatory genes. Among genes which contribute to the attenuated phenotype of NYVAC are *J2R*, *A56R*, *N1L* or *K1L* which have been described to enhance the virulence and replication of VACV *in vivo* [18], [33], [34]. In addition, the absence of other genes which can interfere with host immune responses, such as *M2L*, which are involved in the regulation of inflammatory responses and severity of VACV infection [35], may also contribute to the attenuated phenotype of NYVAC-C7L. Clearly, *C7L* rescues, in part, the *in vivo* replication capacity of NYVAC-C7L providing higher antigen production during infection than NYVAC and, importantly, the vector remains safe.

When we compare the immune responses elicited in mice by NYVAC-B and NYVAC-B-C7L after DNA prime/poxvirus boost immunization approach, the replication-competent NYVAC-B-C7L was clearly superior to NYVAC-B in the induction of specific cellular immune responses against HIV-1 antigens. The data obtained by ELISPOT and ICS revealed that the combination of DNA-B/NYVAC-B-C7L induced T-cell responses mediated by both CD4^+^ and CD8^+^ T cells, were directed to the four vaccine-encoded HIV-1 antigens, and were multifunctional (producing more than one cytokine simultaneously) and durable. NYVAC-B-C7L elicited significantly stronger HIV-1-specific cellular immune responses than replication-restricted NYVAC-B.

The immunological differences between NYVAC-B and NYVAC-B-C7L in the magnitude of vaccine-elicited immune responses could be associated with the kinetics of antigen expression. Bachmann et al. showed that antigen load influences the stimulatory signals provided to T cells by antigen presenting cells (APCs) and subsequently influences the magnitude of the elicited immune response [36]. Other authors have described a high correlation between immunogenicity and *in vitro* expression levels of non-replicating [37], [38], [39], [40] and replicating VACV vectors [41], [42], where high protein expression is necessary for raising good immune responses. Similarly, we have observed that the insertion of *C7L* in the NYVAC-B genome enhanced significantly the antigen production *in vitro* and *in vivo*, correlating to an improvement in the immunogenicity elicited by the replicating NYVAC-B-C7L in comparison to the replication-restricted NYVAC-B. The frequency of total cytokine-producing CD8^+^ T cells was significantly higher in the spleens of animals that were boosted with NYVAC-B-C7L than in those mice boosted with DNA-B/NYVAC-B. Interestingly, the frequency of total cytokines producing CD4^+^ T-cell response was similar in both groups, regardless of the ability of each vector to replicate, indicating that the presence of *C7L* mainly improved the activation of CD8^+^ T-cell responses.

Several studies have emphasized the influence of antigen dose, microenvironment, and specificity of the APCs recruited to the CD8^+^ T-cell activation site in shaping the response by modulating cell function [43]. Considerable evidence suggests that an effective CD8^+^ T-cell response can control or eradicate viral infections. CD8 T cells appear to be particularly important in the immune response to chronic infections and in the long-term control of latent and reactivating viruses [44]. Indeed, potent HIV-1-specific CD8^+^ T-cell responses correlate with acute viral control and long-term nonprogression [5]. The high CD8^+^ T-cell response induced by NYVAC-B-C7L makes this vector an attractive candidate as HIV/AIDS vaccine.

One interesting observation of this study is the change in immunodominance during NYVAC-B or NYVAC-B-C7L immunization. The DNA/NYVAC immunization regimen expressing HIV-1 Env and Gag/Pol/Nef antigens has previously shown an Env-dominant response in animal models and in phase I clinical trials [9], [13], [24], [45]. Our findings indicate that the vaccine regimen using replication competent virus, induced responses that were not dominated by any single vaccine antigen. Thus, the HIV-1-specific immune responses elicited by NYVAC-B-C7L were more balanced in comparison to those induced by parental NYVAC-B, with ENV (36.8% vs 52%), GAG (24.7% vs 18.8%) and GPN (38.4% vs 29.2%). These differences, observed in three independent experiments, could result from increases in response to subdominant epitopes and concomitant decrease in responses to dominant epitopes. Narayan and collaborators have described that heterologous prime-boost immunization incorporating high doses of the second immunogen resulted in peptide-specific CD8^+^ T-cell populations polarized toward a low average functional avidity peptides [46]. Thus, the higher dose of antigen produced in the boost by NYVAC-B-C7L in comparison to NYVAC-B under equal viral loads could explain the shift in response to different antigens. This could have an impact in the vector efficacy, as a balanced immune response targeting epitopes present in Env, Gag, Pol or Nef after vaccination will likely result in more effective inhibition of HIV-1 infection. Preferential targeting of Gag, and the breadth of the Gag response has been correlated with superior viral control in large chronic HIV-1 cohorts, as well as in elite controllers, as demonstrated with a large cohort study in South Africa and in Tanzania [47], [48], [49].

With regard to correlates of protection in HIV-1 infection, although the breadth and magnitude of response to viral antigens are considered important markers in HIV-1 vaccine development, a series of recent studies in mice, non-human primates and humans using multiparameter flow cytometry provide compelling evidence that the quality of the T cell response is a crucial factor in defining a protective T-cell response [50]. In fact, improved control of HIV-1 was associated with increased frequency of multifunctional T cells that produce two or more different cytokines [51], [52], [53]. Multifunctional CD8^+^T cells with enhanced effector functions have been demonstrated after NYVAC vaccination in non-human primates and humans [9], [13], [45], [51]. In our mouse model, both vectors NYVAC-B and NYVAC-B-C7L induced polyfunctional CD4^+^ and CD8^+^ T cells that were mainly IFN-γ^+^TNF-α^+^. Of note, Env- and GPN-specific CD4^+^ and CD8^+^ T-cell responses were also able to secrete some amounts of IL-2. In fact, a high proportion (10-16%) of the HIV-1-specific CD4^+^ T cells expressing simultaneously the three cytokines were found in both immunization groups while the percentages of this population in the CD8^+^ T-cell compartment were lower (1–5%) and only observed in animals boosted with NYVAC-B-C7L, suggesting that good quality of both T-cell responses was induced by the vectors. The presence of polyfunctional CD4^+^ T cells after immunization with both vectors could be favorable for the induction and maintenance of CD8^+^ T-cell memory responses [54]. Indeed, a dysfunctional HIV-1-specific CD4^+^ response is a hallmark of chronic HIV-1 infection, and adversely influences the development and functioning of the CD8^+^ T-cell response [55], [56]. These results might correlate with those previously described in which the functional profile of T cells is directly related to antigen concentration and/or antigen persistence [57], [58].

An important consideration in HIV-1 vaccine development is the establishment of memory responses. Previous studies had shown the induction of long lasting T cell immunity in most of the human volunteers vaccinated with DNA-C prime/NYVAC-C boost [13]. Here, we observed that NYVAC-B-C7L improved the magnitude and quality of the cellular immune responses measured at 53 days post-boost. High levels of IFN-γ and IL-2 were produced in both CD4^+^ and CD8^+^ T-cell compartments after Env stimulation as compared to NYVAC. Hovav and colleagues described that the priming and boosting vectors influenced the magnitude of the secondary CD8^+^ T cell response, but that the ultimate differentiation of these cells to memory cells was exclusively shaped by the second immunogen [59]. In addition, the duration of antigen expression was found to modulate the rate of differentiation of memory CD8^+^ T cells, as this process begins only after most of the antigen is cleared [36], [60]. Furthermore, other factors also appear to influence the evolution of cellular immune memory, and early inflammatory signals delivered by the vector to the immune system have been reported to enhance antigen-presenting activity and control the rate of memory CD8^+^ T-cell development [61]. Thus the differences observed in NYVAC behavior after *C7L* insertion with regard to cytopathic effect, apoptosis induction or viral clearance [19] might have an impact in the type of immune response elicited. Further studies will be needed to determine the innate immunity induced by NYVAC-B-C7L as well as to how the infection impacts on dendritic cells.

Overall, the present data demonstrate that both NYVAC-B and NYVAC-B-C7L in combination with DNA-B are highly immunogenic in mice, induced vigorous and broad T cell responses, comprising of both CD4^+^ and CD8^+^ T cells, which are polyfunctional, and more importantly, these immunization regimens induce long-lasting T cell immunity. Furthermore, we showed that insertion of *C7L* in NYVAC-B genome clearly improves the magnitude and breadth of the cellular immune response induced against the HIV-1 antigens as compared to NYVAC-B. In addition, the replication capability of NYVAC-C7L allows the administration of the vaccine at low and safe doses, thus reducing manufacture burden and making production of NYVAC-C7L vaccines cost effective. Therefore, NYVAC recombinants expressing *C7L* gene are attractive live viral vectors for the development of vaccines and highlight the use of safe attenuated replication competent VACV vectors as improved platform for vaccine development against HIV/AIDS and other infectious diseases.

## Materials and Methods

### Cells and Viruses

Cells were maintained in a humidified air 5% CO_2_ atmosphere at 37°C. African green monkey kidney cells (BSC40) and human cells (HeLa) were grown in Dulbecco's modified Eagle's medium (DMEM) supplemented with 10% newborn calf serum (NCS). 3T3 murine cells were grown in Dulbecco's modified Eagle's medium (DMEM) supplemented with 10% calf serum (CS).

Recombinant viruses with the luciferase gene inserted in the viral thymidine kinase (TK) locus (*J2R*) were used to test virus biodistribution *in vivo*. NYVAC-Luc and WR-Luc recombinants have been previously described [25], [26]. NYVAC-C7L-Luc was generated according to standard methodology using the same plasmid transfer vector used for the generation of WR-Luc and NYVAC-Luc (pSC11-LUC [25]). In addition to *C7L* gene, this new recombinant vector NYVAC-C7L-Luc encodes the luciferase reporter gene under the transcriptional control of the synthetic early/late (E/L) virus promoter.

Parental NYVAC and recombinant NYVAC-B expressing the HIV-1 _Bx08_gp-120 and _III-B_GPN proteins from clade B, kindly provided by Aventis-Pasteur, were previously described [24]. NYVAC-B-C7L was generated using the same plasmid transfer vector used for the generation of NYVAC-C7L (pJR101-C7L [19]). This plasmid directs the insertion of *C7L* gene into the HA locus (*A56R*) of NYVAC-B genome under the transcriptional control of the synthetic early/late (E/L) virus promoter. BSC-40 cells were infected with NYVAC-B recombinant at a multiplicity of 0.01 PFU/cell, and then transfected with 10 µg of pJR101-C7L DNA using lipofectamine reagent according to manufacturer's instructions (Invitrogen). Recombinant NYVAC-B viruses containing the *C7L* gene were selected by consecutive rounds of plaque purification in BSC-40 cells stained with X-Gluc (5-bromo-4-chloro-3-indoxyl-β-D-glucuronidase acid). In addition to *C7L* gene, this new recombinant vector NYVAC-B-C7L contains, as for NYVAC-B, the same cassette of HIV-1 genes in the TK locus (*J2R*) under the transcriptional control of the synthetic early/late virus promoter.

All viruses were grown in BSC-40 cells, similarly purified through two 45% (w/v) sucrose cushions, and titrated by plaque assay in BSC-40 cells. Purity of the recombinants was confirmed by PCR analyses. Throughout the manuscript we will refer to parental NYVAC as replication-restricted and NYVAC-C7L as replication-competent, based on their different abilities to produce progeny virus in cultured human cells, with limited virus replication cycle for the former and complete replication cycle for the latter.

### Analysis of Virus growth

Monolayers of human (HeLa), mouse (3T3) and monkey (BSC-40) cells grown in 12-well tissue culture plates were infected at 0.01 PFU/cell with NYVAC-B, NYVAC-B-C7L and VACV-WR recombinants, at different times postinfection cells were collected with the media, sonicated and virus yields determined by plaque assay in BSC-40 cells as previously described [19].

### Expression of _Bx08_gp-120 and _IIIB_GPN proteins by NYVAC-B and NYVAC-B-C7L in cultured cells

HeLa, 3T3 and BSC-40 cells grown in 12 well-plates were infected at 5 PFU/cell with the recombinants NYVAC-B or NYVAC-B-C7L in presence or absence of adenine arabinoside (AraC, 40 µg/ml, Sigma), an inhibitor of viral DNA replication. At 2, 4, 8 and 24 hours post-infection (hpi), cells were collected and centrifuged at 1500 rpm for 10 min. Cellular pellets were lysed in cold buffer (50 mM Tris-HCl pH 8, 0.5 M NaCl, 10% NP-40, 1% SDS) and samples containing equal amounts of protein were run in 10% SDS-PAGE. The expression of HIV-1 _Bx08_gp-120 and _IIIB_GPN was visualized following western blotting using rabbit polyclonal anti-gp-120 antibody (Centro Nacional de Biotecnologia) and rabbit polyclonal anti-Gag p24 serum (ARP 432, NIBSC, Centralized Facility for AIDS reagent, UK), respectively. Detection of cellular β-actin protein by specific antibody was used as an internal loading control.

### FACS analysis of HIV-1 and VACV viral antigens in cultured human cells

Fluorescent activated cell sorting (FACS) analysis was used to assay the frequency of antigen expressing cells [62]. Monolayers of HeLa cells grown in 6 well tissue culture plates were infected with different doses of NYVAC-B or NYVAC-B-C7L (from 0.01 to 10 PFU/cell). Cultures were harvested at 18 hours post-infection by treating with trypsin. Approximately 10^6^ cells were fixed and permeabilized using cytofix/cytoperm solution (Pharmigen Inc.) according to manufacture's instructions. For detection of viral vaccinia antigen expression, cells were stained using a polyclonal anti-vaccinia antibody (VACV-WR) and then incubated with phycoerythrin conjugated anti-rabbit immunoglobuling (Pharmingen). To test HIV-1 antigen expression we used anti-human immunodeficiency virus (HIV) Gag antibody conjugated to phycoerithrin (clone KC57, Beckman Coulter). A total of 100000 events were acquired on a LSR II FACS (Beckton Dickinson) and analysed using FlowJo software (FlowJo).

### DNA vectors

The two DNA constructs expressing the HIV-1 _BX08_gp-120 (pCMV-_BX08_gp-120) and HIV-1 _IIIB_ Gag-Pol-Nef (GPN) fusion protein (pcDNA-_IIIB_GPN) have been previously reported [24], [63]. Plasmids were purified using Maxi-prep purification kits (Qiagen) and diluted for injection in endotoxin-free phosphate buffered saline (PBS).

### Peptides

The HIV-1 peptide pools Gag-1, Gag-2, Env-1, Env-2, GPN-1, GPN-2, GPN-3 and GPN-4 were provided by the EuroVacc Foundation and were previously described [24]. They spanned the HIV-1 Env, Gag, Pol and Nef regions from clade B included in the immunogens as consecutive 15-mers overlapping by 11 amino acids. For immunological analysis, we grouped the HIV-1 peptides in three main pools: ENV, GAG and GPN. ENV pool comprises Env-1 and Env-2; GAG pool comprises Gag-1 and Gag-2, and GPN pool includes GPN-1, GPN-2, GPN-3 and GPN-4.

### Mice immunization

BALB/c mice were purchased from Harlan. To analyze the biodistribution of virus recombinants in animals, BALB/c were immunized by intraperitoneal route (i.p.) with a dose of 2×10^7^ PFU/mouse of WR-Luc, NYVAC-Luc or NYVAC-C7L-Luc. At different times post-infection mice were sacrificed and mouse tissues were processed for luciferase analysis and plaque assay titration.

To test the immunogeniciy induced by the HIV-1 recombinants, we structured the study around our standard immunization regimen (DNA/Pox combination) which we have previously shown that it elicited strong immune responses to HIV-1 antigens [24], [64]. Animals (n = 4 per group) were primed with 100 µg of DNA-B (50 µg of pCMV-_BX08_gp-120 + 50 µg of pcDNA-_IIIB_GPN) by intramuscular route (i.m.) and two weeks later received an i.p. inoculation dose of 1×10^7^ PFU of NYVAC-B or NYVAC-B-C7L in 200 µl of PBS. Ten days after the last immunization, mice were sacrificed and spleens processed for ELISPOT assay and Intracellular Cytokine Staining (ICS). When long-lived immune response was assayed, the animals (n = 4 per group) were sacrificed at day 53 after the boost. Three independent experiments were performed.

### Measurement of luciferase activity in mouse tissues

Gene expression of recombinant viruses was monitored by the luciferase assay to quantify heterologous gene expression in tissues from mice inoculated i.p with NYVAC-Luc, NYVAC-C7L-Luc or WR-Luc. Tissues were collected at 24, 48 or 72 h post-infection. Peritoneal cells were harvested by mouse peritoneal cavity lavage with 10 ml of sterile PBS, centrifuged at room temperature for 5 min at 1200 rpm and stored at -70°C. At indicated times post-inoculation, animals were sacrificed, spleen, liver, draining lymph nodes and ovaries were dissected under sterile conditions and stored at −70°C. Tissues from individual mice were homogenized in luciferase extraction buffer (300 µl/spleen and 200 µl/ovary, lymph node or peritoneal extracts) (Promega Corp., Madison, Wis.) using an Eppendorf-fitted Dounce homogenizer. Luciferase activity was measured in the presence of luciferin and ATP according to the manufacturer's instructions using a Lumat LB 9501 luminometer (Berthold, Nashua,N.H.), and was expressed as Reference Luciferase Units (RLU) per milligram of protein. Protein content in tissue extracts was measured with a bicinchoninic protein assay reagent kit (Pierce Co., Rockford, Ill.). Tissues from individual mice were collected and homogenized in complete DMEM media to test for the production of infectious virus by plaque assay in BSC-40 cells. The virus titer was expressed as Plaque Forming Units (PFU) per milligram of protein.

### IFN-γ ELISPOT assay

The vaccine- specific cellular immune response in mice was determined using ELISPOT assay measuring the secretion of IFN-γ by splenocytes after stimulation with different peptides pools. Briefly, 10^5^–10^6^ splenocytes (depleted of red blood cells) were plated in triplicate in 96-well nitrocellulose-bottomed plates previously coated with 6 µg/ml of anti-mouse IFN-γ mAb R4-6A2 (Pharmingen, San Diego, CA). HIV-1 peptide pools from clade B and negative control (CTRL) pool were resuspended in RPMI 1640 supplemented with 10% FCS and added to the cells at a final concentration of 5 µg/ml for each peptide. Plates were incubated at 37°C, 5% CO_2_ for 48 h, washed extensively with PBS containing 0.05% of Tween20 (PBS-T) and incubated 2 h at room temperature (RT) with a solution of 2 µg/ml of biotinylated anti-mouse IFN-γ mAb XMG1.2 (Pharmingen, San Diego, CA) in PBS-T. Afterwards, plates were washed with PBS-T and 100 µl of peroxidase-labeled avidin (Sigma, St Louis, Mo) at 1∶800 dilution in PBS-T was added to each well. After 1 h of incubation at RT, wells were washed with PBS-T and PBS. The spots were developed by adding 1 µg/ml of the substrate 3,3′-diaminobenzidine tetrahydrochloride (Sigma, St Louis, Mo) in 50 mM Tris-HCl, pH 7.5 containing 0.015% hydrogen peroxide. The spots were counted with the aid of a stereomicroscope. The responses were given as the number of spot-forming cells per million of splenocytes. In all the cases the background levels were subtracted to each specific peptide pool.

### Intracellular Cytokine Staining (ICS)

The phenotypes of responding T cells were analyzed by ICS and fluorescence-activated cell sorting analysis as described elsewhere [9]. After an overnight rest, 5×10^6^ splenocytes (depleted of red blood cells) were resuspended in RPMI 1640 supplemented with 10% FCS and containing 1 µl/ml Golgiplug (BD Biosciences) to inhibit cytokine secretion. Cells were seeded on M96 plates and stimulated with overlapping peptides that span the entire HIV-1 _BX08_gp-120 and _IIIB_GPN proteins (Gag-pool, Env-pool or GPN-pool) added to the cells at a final concentration of 5 µg/ml for each peptide pool. Cells were incubated at 37°C, 5% CO_2_, and then analyzed by ICS. After 6 h of stimulation, cells were washed, stained for the surface markers, fixed, permeabilized using the BD Cytofix/Cytoperm™ Kit (Becton Dickinson) and stained intracellularly using the appropriate fluorochromes. To analyze the adaptive immune responses (day 10 post-boost), the following fluorochrome-conjugated antibodies were used: CD3-FITC, CD4-Alexa 700, CD8-PerCP, IL-2-PE, IFN-γ-APC and TNF-α-PECy-7. For analyses at day 53 post-boost, the following antibodies were used: CD4-Alexa 700, CD8-FITC, IFN-γ-PECy-7 and IL-2-Alexa-647. All antibodies were from BD Biosciences. Cells were acquired using an LSRII flow cytometer (Becton Dickinson) equipped with a high throughput system. Sample analysis was performed using FlowJo version 8.5.3 (Tree Star, Ashland, OR). The number of lymphocyte-gated events ranged between 10^5^ and 10^6^ dead cells were excluded using violet LIVE/DEAD stain kit (Becton Dickinson). Lymphocytes were gated on a forward scatter area versus side scatter area pseudo-color dot plot and dead cells were removed according to violet LIVE/DEAD staining. To analyze the adaptive immune responses, CD4^+^ and CD8^+^ events (gate previously on CD3^+^ cells) were gated versus IFN-γ, TNF-α and IL-2, and then combined together using the boolean operator. For the analyses at day 53 after the boost, CD4^+^ and CD8^+^ events were gated and the secretion of IFN-γ and IL-2 was analyzed. Data were analyzed using a one-tailed *z* test, to test if the antigen-specific response was higher than the negative control response (response with medium alone).

### Statistical analysis

For the statistical analysis, we have developed a novel approach that corrects measurements for the medium response (RPMI) and at the same time it allows the exact calculation of confidence intervals and p-values of hypothesis tests. For ELISPOT and ICS statistical analysis, it was proceeded as follow. Given the total number of cells, 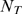, and the number of cells responding to a given antigen, 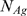, an estimate of the proportion of cells responding to this antigen is given by 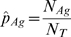. The Bayesian *a posteriori* distribution of 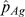 without any *a priori* assumption (i.e., assuming that the true proportion is uniformly distributed between 0 and 1) is the Beta distribution with parameters 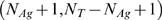 [65]. Let us call 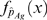 the corresponding probability density function *a posteriori*. Analogously, we can derive the distribution of the proportion of cells responding to RPMI, obtaining the distribution 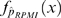. To test whether the antigen response is significantly larger than the RPMI response, we computed the probability density function of the variable 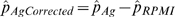 as 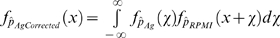. The cumulative density function of this variable is defined in the usual way 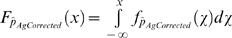. The 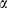 percentile of this variable is defined as 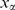 such that 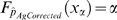. We computed the symmetric 95% confidence interval for the RPMI corrected proportion as 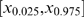. Finally, we consider 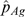 to be significantly larger than 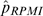 if 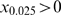. In such a case, 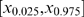 gives the 95% symmetric confidence interval for 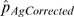. The average 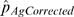 is computed as the expected value of 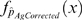 (note that this expected value needs not be in the middle of the confidence interval, 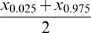).

Whenever two corrected proportions need to be summed, 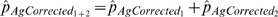, we convolved their probability density functions to obtain the probability density function of the summed proportion, 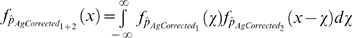, in this way confidence intervals for any sum of corrected proportions can be obtained. Antigen responses were not added unless each component was significantly larger than the corresponding RPMI.

In the ELISPOT experiment, three replicates were obtained for each kind of antigen. The average response to that antigen was computed using only the corrected proportions significantly larger than the corresponding RPMI. The division implied by the averaging process needed a reinterpolation of the probability density function which we carried out using cubic splines as implemented in MATLAB 2008a.

## Supporting Information

Figure S1 Expression of HIV-1 antigens by NYVAC-B and NYVAC-B-C7L vectors in mouse and monkey cells. Western blot showing the kinetics of expression of gp-120 and GPN with time of infection. Murine 3T3 (A) or monkey BSC40 (B) cells were infected with 5 PFU/cell in the absence or presence of 40 µg/ml of cytosine arabinoside, AraC (A), cell extracts collected at various times, analyzed by SDS-PAGE, and Western blots reacted with specific antibodies to Env and GPN. Actin was used as a loading control. M: uninfected mock cells.
(8.71 MB TIF)
