# Supplementary figures and images for: Insertion of Vaccinia Virus C7L Host Range Gene into NYVAC-B Genome Potentiates Immune Responses against HIV-1 Antigens
Source: PLoS One. 2010 Jun 30;5(6):e11406. doi: 10.1371/journal.pone.0011406 (PMC2894869; doi:10.1371/journal.pone.0011406)

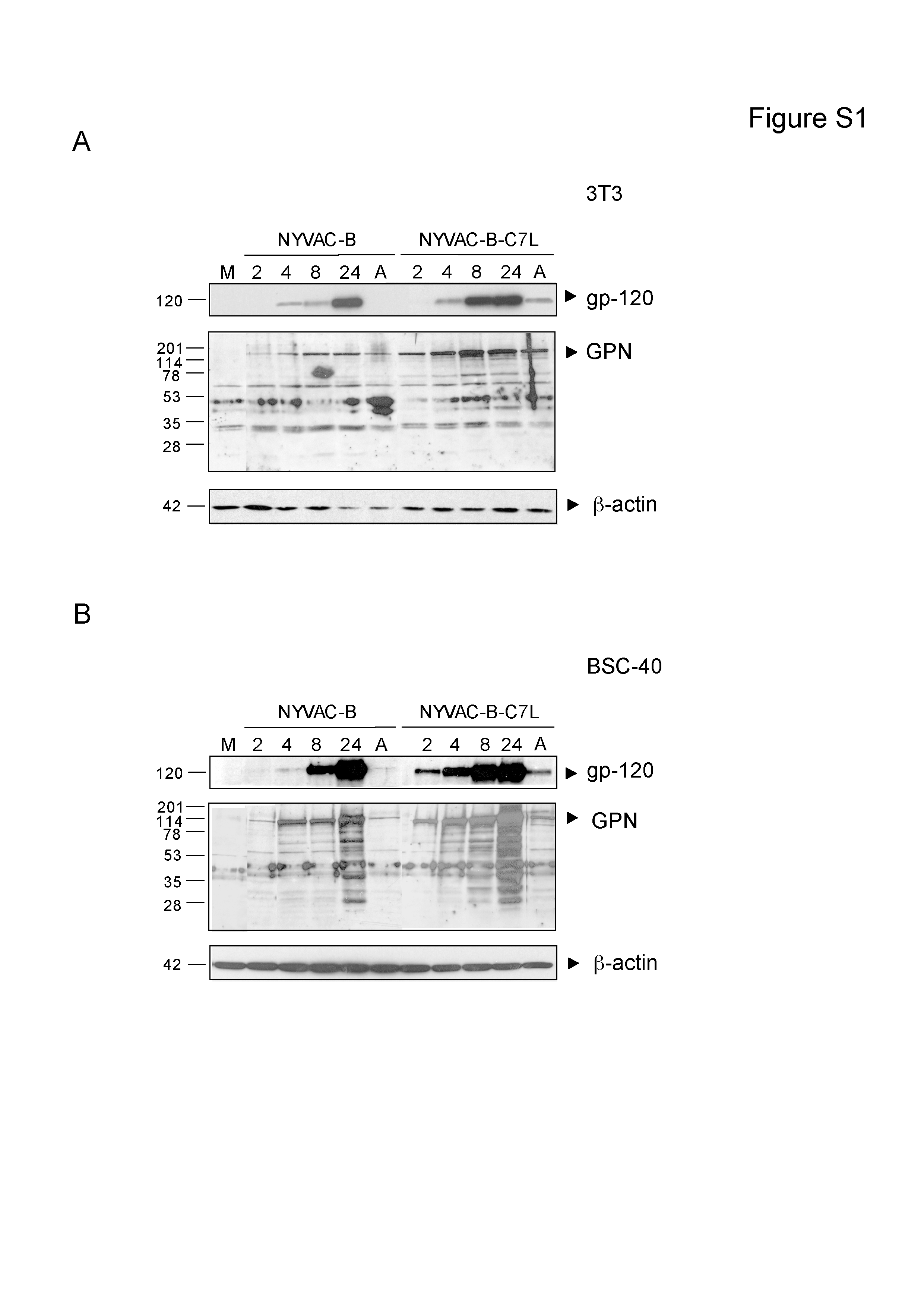

Supplement: Figure S1 — Expression of HIV-1 antigens by NYVAC-B and NYVAC-B-C7L vectors in mouse and monkey cells. Western blot showing the kinetics of expression of gp-120 and GPN with time of infection. Murine 3T3 (A) or monkey BSC40 (B) cells were infected with 5 PFU/cell in the absence or presence of 40 µg/ml of cytosine arabinoside, AraC (A), cell extracts collected at various times, analyzed by SDS-PAGE, and Western blots reacted with specific antibodies to Env and GPN. Actin was used as a loading control. M: uninfected mock cells. (8.71 MB TIF) [file pone.0011406.s001.tif]
